# The Status of Measles and Rubella Outbreak Detection, Early Alerts, and Response in Eastern Mediterranean Region (EMR), 2023

**DOI:** 10.3390/vaccines14030272

**Published:** 2026-03-20

**Authors:** Eman Elmahdy, Eltayeb Elfakki, Amany Ghoniem, Basma M. Saleh, Frank Mahony, Quamrul Hasan

**Affiliations:** 1 World Health Organization, Eastern Mediterranean Region Office, Cairo 11371, Egypt; elmahdye@who.int (E.E.);; 2 School of Science and Engineering, The American University in Cairo, Cairo 11511, Egypt; 3 Independent Epidemiology Consultant, Jakarta 12190, Indonesia

**Keywords:** measles, rubella, outbreak detection, surveillance, genotyping, Eastern Mediterranean Region, immunization, zero dose, timely response, public health preparedness

## Abstract

**Background**: Measles and rubella remain major public health concerns in the Eastern Mediterranean Region (EMR), despite regional elimination goals. In 2023, the region experienced an increase in measles outbreaks. This study assessed outbreak detection and response challenges in either case definition or data analysis, in addition to gaps in laboratory and genotyping data integration to improve preparedness and response. **Method**: A retrospective epidemiological study was conducted using official World Health Organization (WHO) data on measles and rubella (MR) in EMR countries, from 1 January to 31 December 2023. Routine MR surveillance line list, genotyping data and supplemental immunization activity (SIA) reported by countries were used. **Results**: In 2023, 1206 suspected measles outbreaks were reported in 13 countries; 942 (78%) were confirmed. Rubella accounted for 158 confirmed outbreaks. Children under 5 years old comprised 76% of cases, with 62% zero dose. Timely detection was achieved in only 46% of outbreaks, with wide national variation. Genotype B3 predominated, but missing genotyping data limited verification. Six immunization campaigns occurred; however, outbreaks persisted due to high zero dose, limited targeting, and delayed responses. **Conclusions**: Persistent immunity gaps, under detection, inconsistent genotyping, and delayed response hindered MR control in EMR. Strengthening surveillance, integrating epidemiological and molecular data, expanding targeted supplementary immunization activities, and ensuring timely response are essential tasks. Standardized outbreak definitions, capacity building, and regular subnational analyses remain critical to regional elimination goals.

## 1. Introduction

Measles is a highly infectious viral disease primarily affecting the respiratory system before spreading throughout the body. It can cause serious illness, resulting in high morbidity and mortality, particularly in children. Symptoms include high fever, a widespread rash, coughing, and runny nose [1]. Globally, in 2023, there were 669,083 reported measles cases, with an incidence rate of 85.5 cases per 1,000,000 population [2]. Rubella is a contagious viral disease that spreads easily through respiratory droplets from an infected person. It primarily affects children and adults, but when contracted by pregnant women in early pregnancy, there is a 90% chance of transmitting the virus to the fetus, leading to miscarriage, fetal death, or congenital rubella syndrome (CRS) [3]. In 2023, 35,743 cases of rubella were reported globally, with an incidence rate of 4.6 cases per 1,000,000 population [2].

The WHO-EMR includes 22 countries from the Middle East, North Africa, and parts of Central Asia, including the following countries: Afghanistan, Bahrain, Djibouti, Egypt, Iran, Iraq, Jordan, Kuwait, Lebanon, Libya, Morocco, Oman, Pakistan, Qatar, Saudi Arabia, Somalia, South Sudan, Sudan, Syria, Tunisia, United Arab Emirates, Yemen, and the occupied Palestinian territory [4]. In the EMR, the number of reported measles cases initially declined from 89,478 cases in 1998 to 10,072 cases in 2010 (an 89% reduction). However, because of the multiple problems faced by several countries in the region since 2011, progress has slowed; during the period of 2010 to 2019, the number of reported measles cases increased from 10,072 to 33,943 cases [5]. EMR countries reported 90,511 and 1598 confirmed measles and rubella cases, respectively, in 2023. This represents a significant increase from 2022, when 39,266 confirmed measles cases were reported [6].

A case-based measles and rubella surveillance system was established in all EMR countries to ensure the sensitivity and specificity of detection, notification, and investigation of measles- and rubella-suspected cases and outbreaks. The surveillance system has performance indicators such as timeliness of early case detection, completeness of genotyping, and viral detection reporting that assess the transmission of MR and classify the source of cases as imported, import-related, or endemic. Genetic sequencing is used to identify the viral transmission pattern, which is an important complementary for determining endemicity. The identical viral sequencing is considered the same chain of viral transmission or may belong to a different chain transmission. Genotyping is important to know endemic linkage and track importation [5].

Despite the surveillance infrastructure, the EMR witnessed prolonged MR outbreaks, some of which remain ongoing and have extended across multiple years which reflects gaps in surveillance system, delay outbreak confirmation, and inadequate laboratory integration and genotyping, field investigation, and inconsistent implementation of case definitions; these challenges have hindered effective outbreak detection and response. Once an outbreak is confirmed, immediate response actions, including implementing selective or non-selective vaccination based on risk assessment, should be initiated. However, not all outbreak-affected countries initiated response implementation. As shown in Appendix A, only five out of the thirteen countries with measles outbreaks implemented outbreak response activities. Selective vaccination targeted susceptible children and affected areas in low- or medium-risk scenarios, while non-selective vaccination, involving mass vaccination regardless of prior vaccination status, was recommended for high-risk outbreaks to rapidly reduce transmission [7].

Limited studies have been published on the timely detection of outbreaks and response in EMR. Previous reports and WHO bulletins have largely focused on epidemic trends and surveillance indicators, but there is limited analysis of how effectively countries detect and monitor outbreaks. This study addresses this gap by providing a mechanism of measles and rubella outbreak detection across 13 high-risk countries in 2023, focusing on case definition, data analysis, and integration with genotyping data. The aim of the study is to describe the mechanisms of detection and response for MR outbreaks in the EMR, focusing on identifying data analysis challenges, gaps in case definition implementation, and laboratory data integration in order to enhance timely outbreak preparedness and response.

## 2. Materials and Methods

Study design: A retrospective descriptive study was conducted using MR surveillance line list, genotyping data, and supplemental immunization activity (SIA) reports for 2023.

Study Setting: Thirteen countries were selected based on their reported measles and rubella case burden, representing 97% of total regional confirmed cases. Several of the selected countries faced conflicts, population movement, and security challenges that hindered the immunization delivery and transportation of lab samples, in addition to weak infrastructure in the lab capacity and data, and the surveillance system. The following are the high-risk countries: Afghanistan, Djibouti, Iraq, Jordan, Lebanon, Libya, Pakistan, Somalia, Sudan, Syria, and Yemen. And the eliminated-status countries with sporadic cases were Egypt and Iran.

Ethics statement: This study was based on MR surveillance data routinely reported by countries to the World Health Organization-East Mediterranean Region Office (WHO-EMRO). All data were fully anonymized prior to analysis; no personal identifiers were included nor was information allowing individual identification retained. Ethics approval was not required, as the study involved public health routine surveillance data collected under the WHO mandate for disease monitoring and outbreak response. The analysis was conducted in accordance with WHO data protection and confidentiality guidelines.

Data source and data quality: The routine MR surveillance line list data reported by countries to the WHO-EMRO in 2023 (from 1 January to 31 December) provides demographic data, epidemiological data, and molecular genotyping. The number of outbreaks and transmissions are extracted from the cleaned MR line list data. Outbreak immunization response reports sent from countries to the WHO-EMRO included date of supplementary immunization activity (SIA) start date, type of SIA, target age and number, coverage, doses, and type of vaccine. The Measles Nucleotide Surveillance (MeaNS) database, accessed through the WHO Global MeaNS platform. Distinct sequence identifiers (DSIDs) were used to classify the genotyped measles virus strains and determine the transmission chains. Only sequences submitted to and validated within the MeaNS platform were included.

Upon receiving data from countries, immediate data quality checks were conducted by the data management team at the WHO-EMRO to assess timeliness, completeness, and consistency. Feedback was shared with countries promptly to correct the data and resubmit a cleaned version. Only the validated data were included in the analysis of this study.

Data tools: Data was extracted from Excel Power Query sheets and analysis was conducted on the number of suspected cases with EPI and weeks of date onset, and the same with lab confirmed cases. Then, the outbreak case definitions were applied. R Studio 2024.04.2 was employed for the generation of summary tables, graphs, and distribution of cases in EMR.

Data analysis was conducted in three main steps: Firstly, the following outbreak case definitions were applied [7,8]:

Suspected outbreak was defined as five or more suspected cases in a district within 21–28 days. Confirmed outbreak was defined as two or more laboratory-confirmed cases within 21–28 days in the same area, while in eliminated countries, a single confirmed case is considered an outbreak. Ongoing outbreak refers to the continuous occurrence of new cases in a defined area where disease transmission remains active, and surveillance data indicates incidence above expected thresholds despite implemented control measures. Outbreak closure was defined as no new confirmed cases for two incubation periods (8 weeks). The index case was defined as the first case identified during the initial week of an outbreak. The first case appearing on the epidemic curve of a confirmed outbreak is considered the index case in the smallest administrative/geographical area identified by the country. Verification requires a thorough field investigation, including a comprehensive active case search and contact tracing. Secondly, the outbreaks were monitored using the following indicators: timely detection was defined as percentage of measles outbreaks detected within 24–72 h, while timely response refers to the percentage of measles outbreaks for which there is an outbreak response vaccination campaign within 35 days. Finally, assessment of the outbreak situation is performed in the country before and after SIA (observe the status of outbreaks if closed or still ongoing) due to limited data about the timely response [9].

Analytical methods: A comparison analysis was conducted by country (13 high-risk countries) to assess the variation in outbreaks (suspected, confirmed, closed and ongoing) for measles and rubella. The analysis began from basic descriptive epidemiological analysis, including time, person, and place. This is followed by advanced molecular analysis of genotyping data, MeaNS, and DSID. Each unique N450 is given the same sequence ID for all sequences that have the exact match. This can be used to identify identical strains within a country and the chain of transmission. Timely detection is assessed by measuring the onset date of the index case along with the notification date of outbreak. Timely response is evaluated by measuring the interval from outbreak confirmation to the initiation of response vaccination.

## 3. Results

### 3.1. Outbreak Overview by Country

**Figure 1:**
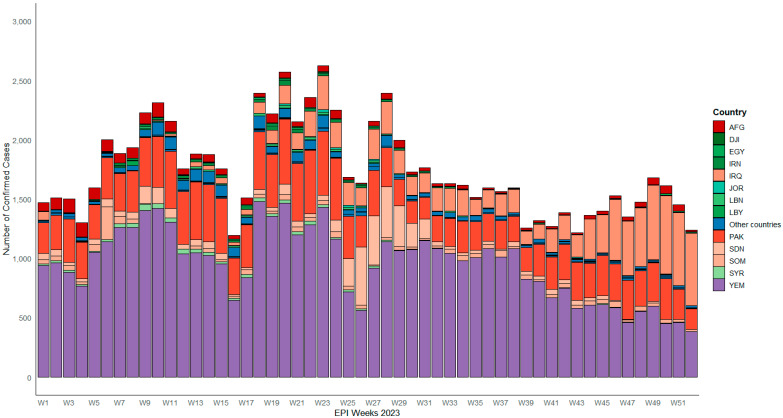
Distribution of measles-confirmed cases by EPI weeks and countries in EMR; own work based on MR surveillance line list database analysis (2023).

In 2023, thirteen countries in the EMR accounted for 97% of all confirmed measles cases in the region. The total suspected measles outbreaks were 1206 across the 13 countries, of which 942 (78%) were confirmed. Rubella accounted for 158 confirmed outbreaks (13%). Sporadic cases included 545 measles and 79 rubella cases. These outbreaks were distributed across 658 districts, with a total of 33,275 cases identified as a portion of the confirmed outbreaks, representing 36% of the 92,083 confirmed MR cases reported, as shown in Table 1. Early detection of outbreaks varied widely by country, ranging from 29% to 100%, as reported by the country. The highest burden of outbreaks throughout the entire period under review occurred in Yemen, Pakistan, and Iraq, which collectively accounted for 85% of total outbreaks in high-risk EMR countries, as shown in Figure 1.

### 3.2. Outbreaks in Eliminated-Status Countries and Source of Infection 

In Egypt and Iran, 243 and 632 confirmed measles outbreaks occurred, respectively, alongside 4 and 13 rubella outbreaks (Table 2). Outbreaks affected 156 districts in these two countries. Egypt reported no endemic MR cases, while Iran reported 1% endemic. Sources of infection were classified as imported, import-related, endemic, and unknown, highlighting ongoing importation risks despite elimination status.

### 3.3. Temporal Trends and Supplementary Immunization Activities (SIAs)

**Figure 2:**
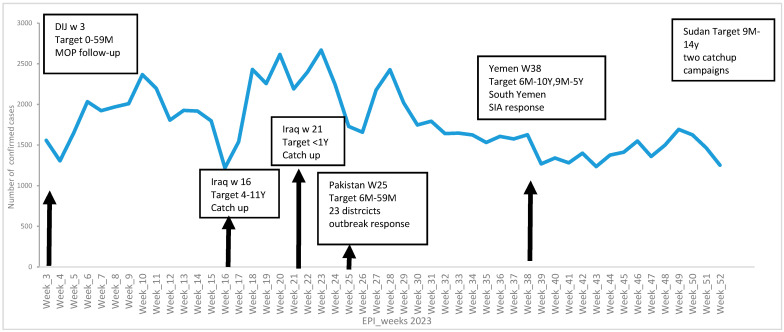
Epi curve of confirmed measles cases in the EMR in high-risk countries (2023) and SIA; own work based on MR surveillance line list and SIA databases analysis.

The number of confirmed measles cases in the EMR surged in 2023, exhibiting multiple peaks. The first peak began between week 2 and week 10 reaching 16,347 cases. A slight decrease was observed in week 11 (2198 cases), followed by another increase during weeks 13 and 14 with 3844 cases. The number of cases showed its magnitude in week 17 to week 24, recording 16,806 cases, and then a minor decline by week 25 (1729 cases). The cases increased again to 10,631 from weeks 27 to 31; from week 32 onward, the number of cases gradually declined to reach 1252 by week 52. SIAs were implemented with varied timing and target age groups, as Djibouti conducted a follow-up SIA at week 1 targeting children under 5 years. Iraq held two catch-up campaigns in weeks 16 and 21 targeting children aged 4–11 years and under 1 year, respectively. Pakistan’s outbreak response campaign took place in week 25, targeting ages 6 months to 5 years across 23 districts. South Yemen and Sudan also implemented outbreak response campaigns targeting children aged 6 months to 10+ years in various weeks (Figure 2). These SIAs may have contributed to outbreak control in some districts; however, many outbreaks persisted.

### 3.4. Age Distribution and Vaccination Status

**Figure 3:**
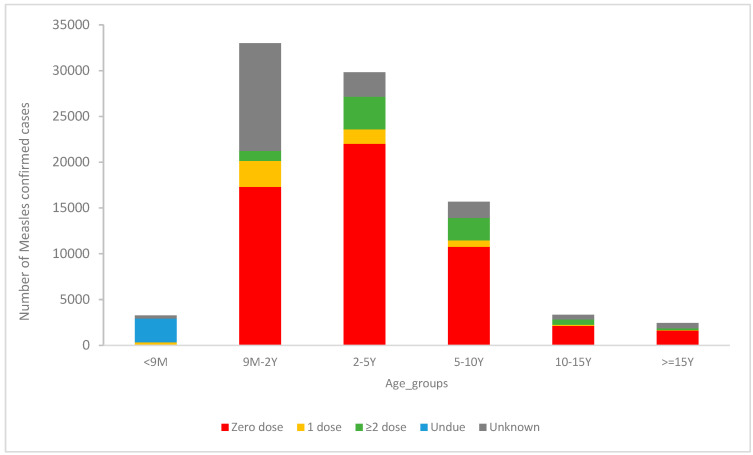
Distribution of confirmed measles cases in high-risk EMR countries by age group and vaccination status; own work based on MR surveillance line list database analysis (2023).

Among confirmed measles cases in high-risk countries, 76% occurred in children under 5 years of age. Zero-dose cases represented 62% of the confirmed cases distributed 25%, 20%, and 12% for 2–5 years, 9 months–2 years, and 5–10 years, respectively (Figure 3). This underscores persistent gaps in young children despite ongoing vaccination efforts.

### 3.5. Genotype Analysis

**Figure 4:**
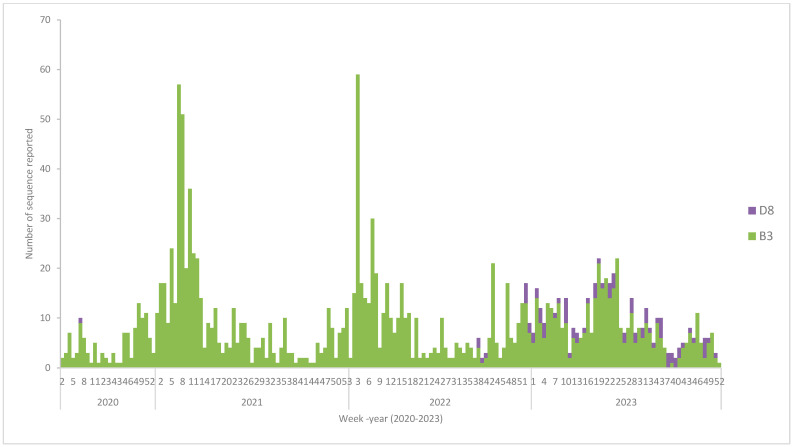
Measles genotypes by week of sample collection WHO-EMR, week 1, 2020 to week 52, 2023. Own work based on MeaNS database analysis.

Between 2020 and 2023, measles virus genotype B3 was the most frequently reported sequence to MeaNS, followed by genotype D8, across ten of the thirteen countries analyzed, namely Afghanistan, Egypt, Iran, Iraq, Jordan, Lebanon, Libya, Pakistan, Sudan, and Syria, as shown in Figure 4. The most commonly reported B3 named strain submitted to the MeaNS database during this period was MVs/Quetta, as shown in Table 3 (PAK/44.20 indicates its widespread circulation in the region). In contrast, genotype D8 was less frequently reported and was more commonly associated with cases involving a documented history of international travel.

### 3.6. Year-on-Year Comparison and Regional Trends

Regarding the case distribution, the number of confirmed measles cases reported in the EMR in 2023 was higher than in 2022, and the percentage of positive cases was 34% in 2023 compared to 32% in 2022, as shown in Appendix B. High-risk countries represented 87% of the total confirmed cases reported from EMR countries, and 34% were positive.

## 4. Discussion

This study applied a descriptive approach to present the early detection of measles and rubella outbreaks in the EMR based on 2023 surveillance data. The findings identify operational gaps in detection mechanisms and data utilization that affect outbreak preparedness.

The majority of reported cases from high-risk countries were part of outbreaks clusters, reflecting gaps in the surveillance, delays in the field investigation aggravated by a lack of security and accessibility constraints, staff turnover, lack of training, and limited lab capacity, in addition irregular data analysis of MR cases and data gaps, like incompleteness and timeliness.

The outbreaks in two eliminated countries reflected the need to intensify the surveillance sensitivity on borders, monitoring international travel and strengthening data integration and genotyping as per the guidelines. Vaccination at country borders is also needed.

The number of confirmed MR cases showed multiple peaks throughout the year. This reflected delayed detection and identified key risk groups for measles cases, highlighting the high zero-dose rate that resulted in immunity gaps. Conflicts, population movements, lack of access to health facilities, and missing catch-up campaigns exaggerated this gap.

The predominance of genotypes B3 and D8 indicates sustained endemic transmission, while some genotype diversity suggests potential importation rather than sustained endemic transmission. This emphasizes the importance of integrating molecular epidemiology and genotyping into outbreak control strategies.

Timely detection and response are essential for outbreak control. In 2023, the percentage of outbreaks detected in a timely manner in the high-risk countries was 46%, which meets the WHO target set for 2026 (40%) [9]. However, some countries showed variability below the target, like Yemen at 32% and Somalia at 29%, which indicated gaps in the surveillance system and delayed outbreak notifications that hindered the outbreak response.

On the other hand, Pakistan achieved a high outbreak detection rate (98%), but the country had delayed response efforts, which started at week 25-2023. This delay in response, in spite of timely detection, highlighted potential gaps in operational readiness, resource mobilization, and effective planning of response activities, which resulted in ongoing outbreaks extending to the next year (2024).

Beyond surveillance timeliness, the coverage and response strategy of the vaccination campaign are other important factors. This is highlighted by the fact that despite six SIAs being conducted in 2023, measles outbreaks persisted into 2024. In Djibouti, a low campaign coverage of 51% likely undermined effectiveness. In Pakistan, the response was limited to only 23 of 152 affected districts, allowing for transmission to continue unabated in uncovered areas. In Yemen, campaigns were restricted to the south, with no activities in the north. Furthermore, Sudan’s campaign was limited to accessible refugee camps due to security constraints, leaving large populations unprotected. Finally, in Iraq, despite two catch-up campaigns, the high case burden at year’s end suggests that the target age groups or geographic scope may have been insufficient to interrupt transmission. Collectively, these factors illustrate that partial or geographically limited responses are inadequate to containing outbreaks, leading to prolonged transmission.

A study on low- and middle-income countries (LMICs) identifying under-resourced health systems noted that variations in measles immunization coverage, due to limitations in the surveillance system and diagnostic capacities, drive outbreaks. Similarly, the extent of ongoing measles outbreaks was influenced by both preventive and control measures, including reactive vaccination campaigns [10].

Along with surveillance timeliness and response coverage, there are other challenges. Sample collection remains a major barrier in some countries (e.g., Yemen) due to inadequate adherence to outbreak confirmation guidelines. Many districts reported multiple suspected outbreaks but only a few were confirmed through laboratory testing. A significant proportion of cases were classified clinically without lab confirmation, affecting data accuracy and potentially leading to under- or overestimation of outbreaks. On the other hand, in laboratory examination and case classification, while sample collection rates are high, a lack of epidemiological links (EPI-links) in countries such as Somalia, Afghanistan, Pakistan, Jordan, and Djibouti limits the ability to establish transmission connections and delays outbreak response. Genotyping data sharing also suffers from major gaps. Yemen, Somalia, and Syria did not share genotype data in 2023, whereas others provided limited updates. This restricted transmission chain identification and molecular epidemiology. In addition, no updates were available on pending cases from 2023 in Afghanistan and Djibouti. This created an uncertainty of outbreak signals and complicated the regular data analysis of surveillance and outbreaks.

Significant data-sharing gaps were another challenge, as countries did not share outbreak investigation reports or line lists with the WHO/EMRO. In addition, incomplete data, particularly the absence of key dates, such as field investigation, SIA request approvals, vaccine shipment, and implementation timelines, limited the ability to accurately calculate the timely response indicator. This represents a key limitation requiring further follow-up. Moreover, silent reporting in several districts that reported zero suspected cases raised concerns about surveillance quality and data accuracy. These underreporting data gaps increase in conflict areas and fragile regions, undermining timely detection of and response to outbreak and masked areas of active transmission.

Comparisons with other countries reflect similar persistent challenges in outbreak detection and response. In Ethiopia, the challenges of outbreak detection and response were reported to be due to a weak surveillance system. This weak system was due to limited budget and logistics, fragile infrastructure, lack of staff training and staff turnover, and absence of EPI monitoring and evaluation [11]. In Indonesia, only 16.7% of investigations met the 48 h timeliness target due to limited resources, lack of training, and data analysis deficiencies [12]. A study on Nepal’s measles outbreak response revealed that a lack of genetic sequencing prevented the identification of transmission links between outbreaks in different geographical areas [13]. The study also highlighted inadequate routine data collection and the absence of a structured framework for monitoring timely response. Finally, it emphasized that SIAs must be planned using epidemiological and population-based surveillance data to accurately determine the target age group based on the distribution of confirmed cases and susceptible populations.

Measles and rubella outbreaks monitoring in non-EMRs, like the region of America (PAHO), achieved measles elimination in 2016, and rubella and CRS elimination in 2015. But from 2018 to 2020, 19 countries experienced measles outbreaks due to importation [14]. In 2023, four outbreaks were reported, affecting 20 jurisdictions, with 49% of reported cases linked to ongoing outbreaks extending into 2024 and 2025. The most affected age groups were children aged 1–9 years and young adults aged 20–29 years. Unvaccinated cases constituted 57% of all cases [15]. Key response actions included strengthening surveillance for rapid case detection, expanding vaccination coverage to close immunity gaps, and conducting rapid immunization activities [16]. In AFRO, nearly half of global high-magnitude outbreaks occurred in 2023, where the case fatality rates increased by 37%. Measles cases increased by 20% in 2023 due to lack of vaccination delivery to poor countries and conflict areas. More than 22 million children missed the first dose of the measles vaccine, resulting in a disruptive measles outbreak reported by 57 countries [17]. Initiatives to combat this include (1) community engagement, such as the Community Epidemic and Pandemic Preparedness Program in Sierra Leone, where trained volunteers used a digital surveillance system for early outbreak detection and rapid response [18]; (2) SIAs, as in Ethiopia, to reach children and reduce cases and deaths [19]; and (3) targeted campaigns in hard-to-reach areas, such as the forests of the Democratic Republic of the Congo [20]. For the European region in 2023, the overall notification rate reported for measles cases was 5.2 cases per million population from 30 EU/EEA. The most affected age group were <1–4 years old. The public health measures in Europe focused on active surveillance, timely field investigation of outbreaks, and emphasizing the achievement of vaccination coverage [21].

Recommendations for MR control in the EMR should be directed towards improving the following gaps: first, the surveillance, by standardizing outbreak definitions and ensuring nationwide implementation, training surveillance officers in outbreak detection and case classification, investigating silent districts and verifying population data and surveillance quality, and strengthening the surveillance system in high-risk countries and condensing the response and immunization areas in fragile and inaccessible areas; second, the genotyping, by integrating surveillance data with genotyping data to ensure all outbreaks are investigated and categorized virologically, and sharing genotyping data from countries and EMR, which is important for distinguishing between endemic and imported sources of transmission; third, the data, by emphasizing the importance of sharing outbreak data, including field investigation reports, line lists, genotypes, and response actions, and regular analysis of weekly data on the district level for early detection of peaks and clusters, which can accelerate the response and improve preparedness; fourth, monitoring of outbreaks and response strategies, by expanding age eligibility in high-transmission zones, using digital dashboards for real-time monitoring, improving campaign quality via rapid post-campaign assessments, and engaging humanitarian actors for outreach in inaccessible zones; finally, vaccination, by prioritizing zero-dose populations and mobile, migrant groups, strengthening routine immunization, and integrating outbreak preparedness.

As part of ongoing efforts and measures taken by EMRO, webinars were organized with countries to orient them on how to apply the case definition of outbreaks to detect the outbreaks. Furthermore, training was conducted on how to detect outbreaks early through data management and immunization. Lastly, the MR outbreak dashboard was designed to regularly visualize outbreak alerts and identify the ongoing outbreaks.

## 5. Conclusions

Despite comprehensive outbreak response efforts, EMR countries continue to face significant challenges in their early detection of and response to outbreaks. Persistent zero-dose burden, surveillance gaps, lack of regular data analysis, incomplete data, and limited genotyping capacity, with silent reporting in some areas, hinder timely detection and effective response to outbreaks. Strengthening case-based surveillance, regular analysis of weekly reports (by time, person, and place), integrating case-based data with genotyping and cluster analysis, and conducting retrospective investigations in silent districts are essential tasks for a robust outbreak verification and response. Countries should focus on establishing well-functioning case-based MR surveillance systems that align with regional guidelines and performance indicators. This includes ensuring prompt case investigation, active case finding in low-coverage areas, genotyping, and leveraging epidemiological data to guide immunization strategies and SIA planning. Future research should explore immunity gap modeling and forecasting clusters, epidemic cycles, and outbreaks areas, based on the immunity profile, susceptibility, and coverage.

## Figures and Tables

**Table 1:** Number of measles and rubella outbreaks in high-risk countries EMR; own work based on MR surveillance line list database analysis (2023).

Country	Suspected Outbreaks	Confirmed Measles Outbreaks	Ongoing Measles Outbreaks	Confirmed Rubella Outbreaks	Sporadic Measles	Sporadic Rubella	Districts with Outbreaks	Measles Outbreak Cases	Early Detection of Outbreaks
Afghanistan	177	217	21	23	154	23	125	2348	72%
Djibouti	9	8	0	1	0	1	6	72	57%
Iraq	141	138	16	2	65	0	83	6630	48%
Jordan	9	5	1	0	3	3	5	120	100%
Lebanon	15	13	0	1	16	2	14	324	100%
Libya	43	14	2	22	24	22	12	62	50%
Pakistan	214	214	102	63	26	8	152	15,945	98%
Somalia	36	43	12	3	24	0	30	1772	29%
Sudan	61	39	0	21	40	8	37	444	53%
Syria	68	58	1	2	67	12	38	665	57%
Yemen	433	193	0	20	126	0	156	4893	32%
Total	1206	942	140	158	545	79	658	33,275	46%

**Table 2:** Number of measles and rubella outbreaks in eliminated countries (Egypt and Iran) and source of infection in EMR based on our own work on MR surveillance line list database analysis (2023).

Country	Suspected Outbreaks	Confirmed Measles Outbreaks	Confirmed Rubella Outbreaks	Districts with Outbreaks	Number of Outbreak Cases	Source of Infection
Egypt	3508	243	4	56	260	131 (53%) imported, 110 (45%) import-related, 6 (2%) unknown
Iran	7979	632	13	100	658	190 (30%) imported, 329 (51%) import-related, 118 (18%) unknown, 8 (1%) endemic

**Table 3:** Most frequent B3 and D8 genotype name strains reported to MeaNS database in the selected countries, from week 1, 2020, to week 52, 2023. Own work based on lab database analysis.

B3 Name Strain/Country	DSID (Distinct Sequence Identifier)	2020	2021	2022	2023
MVs/Alburaimi.OMN/15.20	6382	5	15	12	22
Afghanistan				2	
Pakistan		2	15	10	21
MVs/Bradford.GBR/13.18	5258	11	26	6	
Iran, Islamic Republic of			1		
Pakistan		11	25	6	
MVs/Islamabad.PAK/1.13	4194		2	1	
Iran, Islamic Republic of			1	1	
Pakistan			1		
MVs/Kabul.AFG/20.14/3	4298	65	81	19	
Afghanistan				1	
Pakistan		65	81	18	
MVs/Kohistan.PAK/51.20	6464	5	39	9	
Pakistan		5	39	9	
MVs/Oslo.NOR/16.18	5287	1		2	3
Afghanistan				1	
Pakistan		1		1	3
MVs/Quetta.PAK/44.20	6418	2	70	109	99
Afghanistan				6	
Egypt					
Iran, Islamic Republic of			2	44	25
Jordan					1
Pakistan		2	68	58	72
Lebanon				1	1
D8 Name strain/Country	DSID	2020	2021	2022	2023
MVs/Almaty.KAZ/10.23	8491				5
Egypt					5
MVs/Frankfurt Main.DEU/17.11	2266			5	10
Iraq					2
Syrian Arab Republic				5	8
MVs/Rudaki.TJK/49.21	8248			1	9
Egypt				1	9

## Data Availability

The original line list data supporting the conclusions of this article were provided to the World Health Organization by member states under agreements of confidentiality for public health purposes. Aggregated datasets are available from the corresponding author upon reasonable request. Genotyping data are publicly available in the WHO measles nucleotide surveillance (MeaNS) database.

## Abbreviations

The following abbreviations are used in this manuscript:

AFRO	African Regional Office (WHO)
CDC	Centers for Disease Control and Prevention
CFR	Case Fatality Rate
CRS	Congenital Rubella Syndrome
DSID	Distinct Sequence Identifier (used in MeaNS)
DRC	Democratic Republic of the Congo
ECDC	European Centre for Disease Prevention and Control
EMRO	Eastern Mediterranean Regional Office (WHO)
EPI	Expanded Programme on Immunization
EPI-link	Epidemiologically Linked
LMICs	Low- and Middle-Income countries
MeaNS	Measles Nucleotide Surveillance (WHO online database)
MR	Measles and Rubella
PAHO	Pan American Health Organization
RCC	Regional Certification Commission (for Measles/Rubella Elimination)
SIA	Supplementary Immunization Activity
WHO	World Health Organization

## Appendix A. Summary Table Shows Outbreak Response Plans and Its Implementation by High-Risk Countries

**Country Name**	**Plans of Outbreak Response in 2023**
Afghanistan	No
Djibouti	Yes, follow-up campaign was conducted
Iraq	Yes, two catch-up campaigns
Jordan	No
Lebanon	No
Libya	No
Pakistan	Yes, outbreak response campaign
Sudan	Yes, two catch-up campaigns
Somalia	No
Syria	No
Yemen	Yes, outbreak response campaign
Egypt	No
Iran	No
Country name	Plans of outbreak response in 2023
Afghanistan	No
Djibouti	Yes, follow-up campaign was conducted
Iraq	Yes, two catch-up campaigns
Jordan	No
Lebanon	No
Libya	No
Pakistan	Yes, outbreak response campaign
Sudan	Yes, two catch-up campaigns
Somalia	No
Syria	No
Yemen	Yes, outbreak response campaign
Egypt	No
Iran	No

## Appendix B. Distribution of Suspected, Confirmed, and Positivity of MR, 2022–2023, in High-Risk Countries in the EMR; Own Work Based on Line List Analysis

**Country**	**Suspected_MR_2022**	**Tested_MR_2022**	**Positive_Measles_2022**	**Percent_Positive_2022**	**Suspected_MR_2023**	**Tested_MR_2023**	**Positive_Measles_2023**	**Percent_Positive_2023**
Afghanistan	8933	5181	8904	5153	5087	2488	57	48
Djibouti	289	114	287	113	188	73	66	65
Egypt	2978	3508	2974	3487	108	357	4	10
Iran	10,620	7979	10,146	7879	228	760	2	10
Iraq	592	12,191	568	4386	23	1932	4	44
Jordan	468	1365	462	1365	31	162	7	12
Lebanon	155	741	76	158	8	91	11	58
Libya	533	1966	518	1910	32	198	6	10
Pakistan	17,737	37,192	15,616	35,528	6316	16,009	40	45
Sudan	3406	5308	1885	1093	632	419	34	38
Somalia	1304	3136	1304	3136	792	1784	61	57
Syria	1224	2015	1218	1933	219	666	18	34
Yemen	27,354	51,450	3869	3848	1824	2241	47	58
Total	75,593	132,147	47,827	70,010	15,488	27,180	32	39
Percent of high-risk countries	95	92	93	87	98	90	30	34
